# The Effect of Diabetes Mellitus on Central Corneal Thickness Values: A Systematic Review and Meta-Analysis

**DOI:** 10.3390/ijms26178695

**Published:** 2025-09-06

**Authors:** Arda Uzunoglu, Juan José Valenzuela-Fuenzalida, Karin Morales-Calderón, Isidora Aguilar-Aguirre, Alejandro Bruna-Mejias, Pablo Nova-Baeza, Mathias Orellana-Donoso, Gustavo Oyanedel-Amaro, Alejandra Suazo-Santibañez, Juan A. Sanchis-Gimeno, Jose E. León Rojas, Guinevere Granite

**Affiliations:** 1 Republic of Turkey Ministry of National Education, Kocaeli 06420, Türkiye; arda-uzunoglu@hotmail.com; 2 Departamento de Morfología, Facultad de Medicina, Universidad Andrés Bello, Santiago 8370146, Chile; juan.kine.2015@gmail.com (J.J.V.-F.); kisimorales@gmail.com (K.M.-C.); isiaguilar427@gmail.com (I.A.-A.); alejandro.bruna@upla.cl (A.B.-M.); pablo.nova@usach.cl (P.N.-B.); 3 Departamento de Ciencias Química y Biológicas, Facultad de Ciencias de la Salud, Universidad Bernardo O’Higgins, Santiago 8370993, Chile; 4 Department of Morphological Sciences, Faculty of Medicine and Science, Universidad San Sebastián, Santiago 7510157, Chile; mathor94@gmail.com; 5 Escuela de Medicina, Universidad Finis Terrae, Santiago 7501015, Chile; 6 Facultad de Ciencias de la Salud, Universidad Autónoma de Chile, Santiago 8370458, Chile; g.oyanedelamaro@gmail.com; 7 Faculty of Health and Social Sciences, Universidad de Las Américas, Santiago 8370040, Chile; alej.suazo2025@gmail.com; 8 GIAVAL Research Group, Department of Anatomy and Human Embryology, Faculty of Medicine, University of Valencia, 46001 Valencia, Spain; juan.sanchis@uv.es; 9 Cerebro, Emoción y Conducta (CEC) Research Group, Escuela de Medicina, Universidad de las Américas (UDLA), Quito 170135, Ecuador; 10 Department of Surgery, F. Edward Hébert School of Medicine, Uniformed Services University of the Health Sciences, Bethesda, MD 20814, USA; guinevere.granite@usuhs.edu

**Keywords:** central corneal thickness, CCT, diabetes mellitus, DM2, DM1, corneal disease

## Abstract

Diabetes mellitus (DM) is a chronic metabolic disorder that can induce systemic and ocular complications. Among the latter, an increase in central corneal thickness (CCT) has been reported, potentially affecting endothelial function and increasing the risk of ocular disease. This study aimed to determine the impact of DM on CCT and to assess its correlation with diabetes duration and glycosylated hemoglobin (HbA1c) levels. A systematic literature search was conducted in Web of Science (1980–2025) following a PICO-based strategy. Observational studies evaluating CCT in diabetic patients were included. Data were analyzed using a random-effects model. Statistical heterogeneity was assessed with χ^2^ test, *p* values, and I^2^ index. Publication bias was evaluated using Begg’s funnel plot and Egger’s regression test. Twenty-nine studies were included in the meta-analysis. Diabetic patients showed significantly higher CCT values compared to controls, particularly in those with long-standing DM (*p* < 0.001) and poor glycemic control (HbA1c, *p* < 0.001). Egger’s regression suggested an association between increasing CCT, disease duration, and HbA1c levels, while funnel plot asymmetry indicated potential publication bias. CCT appears to increase in patients with long-term DM and inadequate glycemic control. These findings highlight the relevance of CCT assessment as a potential indicator of corneal changes in diabetic patients.

## 1. Introduction

Diabetes mellitus (DM) is a chronic metabolic disorder characterized by impaired metabolism of carbohydrates, lipids, and proteins due to defects in insulin secretion, insulin action, or both. Persistent hyperglycemia leads to long-term damage of multiple organs, including the kidneys, nerves, heart, blood vessels, and ocular structures [1]. The global prevalence of DM is estimated at 6.4%, with a marked increase in cases during the last two decades [2]. The risk of complications rises with longer disease duration and inadequate glycemic control [3].

In the eye, DM can affect virtually all structures. Corneal alterations, such as epithelial fragility, recurrent erosions, reduced sensitivity, impaired epithelial and endothelial barrier function, and stromal edema, are well documented [4,5]. Collectively, these manifestations are referred to as diabetic keratopathy, which is reported in up to 70% of diabetic individuals [6].

Central corneal thickness (CCT) is a clinically relevant parameter, routinely assessed in refractive surgery screening [7], corneal dystrophy and ectasia detection [8,9], dry eye management [10], and contact lens tolerance [11,12]. Since CCT depends on endothelial function, it also serves as an indirect marker of corneal health and may reflect systemic conditions such as DM [13].

Several studies have reported increased CCT in patients with diabetes [3,14,15,16,17,18,19,20,21,22,23,24,25,26,27,28]. Some investigations suggest this thickening correlates with longer disease duration [16,20,25] or higher glycosylated hemoglobin (HbA1c) levels [17,21,23]. However, other studies have failed to confirm these associations [3,14,15,18,19,22,24,26,27,28], leaving the evidence inconsistent.

The aim of this study was to perform a systematic review and meta-analysis to evaluate the impact of DM on CCT and to clarify whether disease duration and glycemic control (HbA1c) are associated with CCT alterations.

## 2. Methods

### 2.1. Protocol and Registration

This systematic review and meta-analysis followed the PRISMA 2020 guidelines [29,30,31,32]. The study protocol was prospectively registered in the International Prospective Register of Systematic Reviews (PROSPERO; registration number: CRD42024574548), https://www.crd.york.ac.uk/prospero/.

### 2.2. Search Strategy

A comprehensive literature search was conducted in July 2025 across MEDLINE (via PubMed, National Library of Medicine, Bethesda, MD, USA), Web of Science (WOS, Clarivate, Philadelphia, PA, USA), Scopus (Elsevier, Amsterdam, The Netherlands), Cumulative Index to Nursing and Allied Health Literature (CINAHL, EBSCO, Ipswich, MA, USA), and Google Scholar (Google LLC, Mountain View, CA, USA), covering publications from the database inception until July 2025. 

The search strategy combined controlled vocabulary (e.g., MeSH terms) and free-text words related to diabetes and corneal thickness. Boolean operators (AND/OR/NOT) were applied. An example of the PubMed search strategy is provided in Supplementary Table S1.

Search terms included:

- “Central corneal thickness” OR “CCT”
- “Diabetes mellitus” OR “type 1 diabetes” OR “type 2 diabetes”
- “Corneal disease”

### 2.3. Eligibility Criteria

Studies were included if they met the following PICO framework:

- Population: Adults with diabetes mellitus (type 1 or type 2);
- Intervention/Exposure: Diabetes mellitus with varying disease durations or glycemic control (HbA1c);
- Comparison: Non-diabetic individuals or diabetic patients with better metabolic control;
- Outcomes: Central corneal thickness (CCT) measurements.

We included observational studies (cross-sectional, cohort, case–control) and case series. No language or publication date restrictions were applied. Studies were excluded if they lacked quantitative CCT data or if DM diagnosis was not clearly established.

### 2.4. Study Selection

Three reviewers independently screened the titles and abstracts of retrieved records. Full texts of potentially relevant articles were evaluated against eligibility criteria. Disagreements were resolved by consensus or by consulting a third reviewer.

Inter-reviewer agreement was assessed using Cohen’s kappa statistic (κ = 0.77), indicating good agreement.

### 2.5. Data Collection Process

Two authors independently extracted data using a standardized form. Extracted variables included:

- First author and publication year;
- Country/region of study;
- Population characteristics (age, sex, sample size, type of diabetes);
- Duration of diabetes and HbA1c levels;
- CCT values (mean, SD, measurement method);
- Study design and clinical considerations.

### 2.6. Risk of Bias Assessment

Methodological quality was assessed using the ROBINS-I tool, which evaluates bias in non-randomized studies of interventions. Domains assessed included confounding, participant selection, measurement of interventions and outcomes, missing data, and selective reporting.

### 2.7. Statistical Methods

All analyses were performed using R statistical software (version 5.1; accessed October 2024). Pooled estimates of CCT differences between diabetic and control groups were calculated using the DerSimonian–Laird random-effects model. Proportions were stabilized using the Freeman–Tukey double arcsine transformation.

Heterogeneity was assessed with the I^2^ statistic and interpreted with 95% confidence intervals as:

0–40%: low heterogeneity

30–60%: moderate

50–90%: substantial

75–100%: considerable

Publication bias was evaluated with funnel plots and Egger’s test.

### 2.8. Subgroup Analysis

Pre-specified subgroup analyses were conducted according to:

Type of diabetes (DM1 vs. DM2);

Duration of diabetes (<10 years vs. ≥10 years);

Glycemic control (HbA1c < 7% vs. ≥7%);

Geographical region;

Sex distribution;

Sensitivity analyses were performed by excluding studies at high risk of bias and by comparing fixed- vs. random-effects models.

## 3. Results

### 3.1. Included Studies

**Figure 1:**
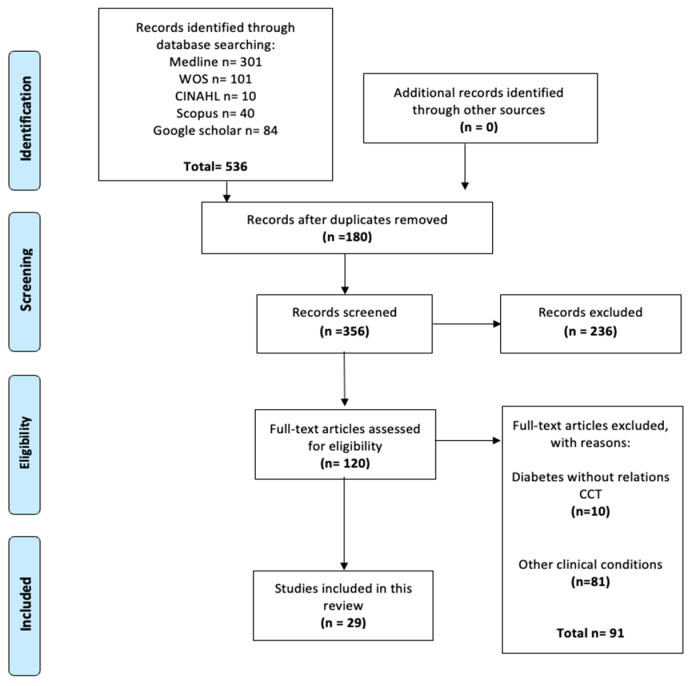
A flow diagram of the search strategy.

The database search provided 536 publications for further assessment and a full-text review. Among them, 507 studies were excluded for various reasons: inadequate database, non-human studies, or subjects undergoing prior corneal surgery. Finally, 29 studies met the inclusion criteria and were included in this meta-analysis. The detailed process of data selection is described in Figure 1 and Table S1.

### 3.2. Characteristics of Included Studies

The total number of included studies was 29 [3,14,15,16,17,18,19,20,21,22,23,24,25,26,27,28,33,34,35,36,37,38,39,40,41,42,43,44,45], and the total number of patients was 7622. Geographic regions included: Asia with a total of 12 papers [20,21,22,26,33,34,35,36,37,39,42,44], Europe with 14 articles [3,14,15,16,18,19,23,24,25,27,28,41,43,45], and the United States of America with 3 studies [17,38,40]. Non-reported sex/total n 5138 reportados, 2484 non-reported. Male and female (29 artícles): 2403 male and 2735 female and mean age: 55.8 (Table 1).

### 3.3. Anatomy and Histology of the Eye and Cornea

The human eye is attached to the orbit by six extrinsic muscles, which also control its movement: levator palpebrae superioris, superior rectus, inferior rectus, medial rectus, lateral rectus, inferior oblique, and superior oblique muscles. The eye is made up of three structural layers: (1) the outer layer or tunica fibrous, (2) the middle layer or tunica vascular, and (3) the nervous layer or retina. The fibrous tunica of the eye comprises two main parts: the sclera, which corresponds to the white portion, and the cornea, which is transparent. It is composed of connective tissue rich in collagen fibers, which are cross-linked and arranged parallel to the ocular surface. This structure provides support, maintaining the shape and consistency of the eyeball [16]. The vascular tunic of the eye is made up of the choroid, the stroma of the ciliary body, and the iris. It is a highly vascularized layer, composed of loose connective tissue, abundant blood capillaries, and collagen and elastic fibers, as well as cells with melanin [3]. Its main function is to provide essential nutrients to the retina. Finally, the retina is made up of three main parts: the external pigment epithelium, the internal nerve retina, and the epithelium of the ciliary body and iris. As the photosensitive layer of the eye, the retina is directly connected to the central nervous system via the optic nerve, which transmits visual information to the brain’s visual cortex for processing [46]. The cornea is made up of three cell layers, separated by two key membranes. The first layer, the corneal epithelium, is a flat stratified epithelium without a stratum corneum, which is continuous with the conjunctival epithelium that covers the adjacent sclera. Between the corneal epithelium and the underlying stroma is the Bowman’s membrane, which functions as a barrier against the spread of infection and lacks regenerative capacity. The corneal stroma is composed of lamellae formed by parallel bundles of collagen fibers, responsible for the transparency of the cornea. On the other hand, Descemet’s membrane, which constitutes the basal lamina of the corneal endothelium, is an interwoven network of fibers and pores that separates the endothelium from the adjoining corneal stroma. The last layer, the corneal endothelium, is a monolayer of flattened cells that lines the inner surface of the cornea and delimits the anterior chamber of the eye [46].

### 3.4. Statistical Results

The characteristics of the studies included in this review and meta-analysis are shown in Table 1. The 29 studies were published between 1980 and 2023. Sample sizes in these studies range from 20 to 1191. The method to determine CCT values varied across the studies, including Haag-Streit pachymeter, digital pachymeter, specular microscopy, ultrasonic pachymeter, Scheimpflug camera, biopachymeter, non-contact specular microscopy, non-contact corneal topography, and high-resolution (HR) Pentacam topography. Based on studies included in this systematic review and meta-analysis, there are controversial results on increased CCT values in diabetic patients compared to control subjects. Sixteen studies observed that CCT values increased in diabetic patients compared to control subjects, and there are seven studies in which this correlation was not observed. As for the diabetes duration effect on CCT values, there were three studies in which CCT values increased because of that, and ten studies in which diabetes duration did not alter CCT values. Another correlation was observed in diabetic patients: three studies showed that CCT values increase in regard to HbA1c levels, and ten studies did not observe this correlation.

The data the group was based on were CCT values according to duration of diabetes, HbA1c levels, and age range, respectively. Based on the data in Table 2, it can be stated that the largest number of studies are in the diabetes duration range of between 6.0 and 10.9 years. Those that present an average of the highest level of CCT, however, are between 11 to 15.9 years, which may be explained by the following. The longer they suffer from diabetes, the higher the level of sorbitol that can accumulate in the lens, due to the dysfunction of the endothelial cells of the cornea, increasing the CCT with respect to physiological ranges. This range in relation to CCT levels presented a mean of 556.82, which did not show statistically significant differences between years of diabetes duration and CCT *p* = 0.312 (Table 2 and Table 3). Regarding Table 3, the highest mean CCT (558.5) is described in a range of 9.1 to 10.5 HbA1c levels, including three articles, indicating that a high level of glycated hemoglobin can be related to a high level of glucose in the blood (Table 3). Conversely, there are at least eight papers that do not mention the measurement of HbA1c in the patients in the study. Finally, in Table 4, the largest number of articles is in a range between 51 and 60 years old with an average CCT of 556.09, while the highest average CCT is 568.98 and corresponds to a range between 41 and 50 years old, which includes five articles (Table 4).

Table 5 presents descriptive statistics with variables such as mean age, months diagnosed with type 2 diabetes mellitus, HbA1c levels, and CCT. For each of these variables, measures of central tendency, dispersion, and normality are presented. The number of subjects analyzed (N) is 29 for all variables. The mean age is 42.7 years, with a standard error of 3.83, while the 95% CI for the mean is between 34.8 and 50.5 years. The median age is 48.2 years, with a standard deviation of 20.6, a variance of 425, and a range of 72.1 years, with minimum and maximum values of 0 and 72.1, respectively. The age distribution asymmetry is −0.911 with a standard error of asymmetry of 0.434, while kurtosis is −0.072 with a standard error of 0.845. The Shapiro–Wilk test of normality yields a W value of 0.897 with a *p*-value of 0.008, indicating that the variable does not follow a normal distribution. As for the months with a diagnosis of type 2 DM, the average is 131 months, with a standard error of 15.4. The 95% CI for the mean ranges from 99.6 to 163 months. The median is 130 months, and the standard deviation is 82.8. The variance of this variable is 6863, and the range is 311 months, with a minimum value of 0 and a maximum value of 311 months. The distribution skewness is 0.222, with a standard error of 0.434, while kurtosis is −0.388 with a standard error of 0.845. The Shapiro–Wilk test shows a W-value of 0.969 and a *p*-value of 0.532, indicating that the distribution does not deviate significantly from normal. Regarding HbA1c levels, the mean is 5.66 with a standard error of 0.754 and a 95% CI between 4.11 and 7.20. The median is 7.30 with a standard deviation of 4.06. The variance is 16.5, while the range is 10.7, with minimum and maximum values of 0 and 10.7, respectively. The distribution skewness is −0.545 with a standard error of 0.434, while kurtosis is −1.37 with a standard error of 0.845. The Shapiro–Wilk test shows a W-value of 0.806 and a *p*-value of less than 0.001, indicating a significant deviation from normal. Finally, the CCT has a mean of 553 μm, with a standard error of 4.07 and a 95% CI between 544 and 561. The median is 552 μm, with a standard deviation of 21.9 and a variance of 480. The range is 95.5, with minimum and maximum values of 515 and 610 μm, respectively. The distribution skewness is 0.491, with a standard error of 0.434, while kurtosis is 0.303 with a standard error of 0.845. The Shapiro–Wilk test shows a W-value of 0.975 and a *p*-value of 0.708, indicating that this variable follows a normal distribution (Table 5).

### 3.5. Robins Risk of Bias

Regarding risk of bias, this was assessed using Robins. For the Confounding item, 22 studies had a low risk of bias, and 7 studies had a high risk of bias. For the Selection item, 17 studies had a low risk of bias, 11 studies had a high risk of bias, and 1 study was classified as unclear. Regarding the Intervention Measurement item, 27 studies presented a low risk of bias, 1 study presented a high risk of bias, and 1 study was classified as unclear. For the Missing item, 22 studies presented a low risk of bias, 2 studies presented a high risk of bias, and 1 study presented an unclear risk. For the Measurement of Outcomes item, 23 studies presented a low risk of bias, and 6 studies presented a high risk of bias. For the Reported item, 26 studies presented a low risk of bias, and 3 studies presented a high risk of bias. Finally, overall, 19 studies presented a low risk of bias and 10 studies presented a high risk of bias (Table 6).

### 3.6. Clinical Considerations

Patients with DM experience significant alterations in the anatomy and functionality of the cornea, which can affect long-term visual health if the problem is not treated. One of the most relevant variations observed in different studies is the increase in CCT [14,15,24]. In addition, morphological modifications, such as polymegathism, pleomorphism, and reduction in endothelial cell density, have been recognized, as well as increased endothelial permeability [17,38,40]. These changes are significant when HbA1c levels are elevated, as well as when glycemic control is present and corneal health care is deficient [3,23,33,41]. This is reflected in the structural vulnerability of the corneal endothelium and its relationship with systemic complications, such as diabetic retinopathy. This is not only harmful to people with diabetes, but it also presents an increased risk of complications during intraocular surgical procedures, such as cataract surgery, due to the increased susceptibility of the corneal endothelium to metabolic and mechanical damage [19,20,36,41]. This is why it is essential to perform continuous monitoring using specular microscopy in addition to making evaluations of parameters, such as HbA1c levels and albuminuria, to prevent complications.

The use of viscoelastic devices, the implementation of precise surgical techniques, and the reduction of operative times are recommended options to minimize endothelial damage in patients. These observations reinforce the importance of comprehensive clinical follow-up to preserve corneal health and prevent long-term visual complications [33].

## 4. Discussion

Several studies have described corneal alterations in patients with DM, with increased CCT being one of the most reported. In general, disease duration and glycemic control appear to be key determinants of these changes. Busted et al. [14] had already suggested that increased CCT could represent an early change in the diabetic eye, associated with endothelial dysfunction and increased corneal hydration. Subsequently, Larsson et al. (1996) [16], Roszkowska et al. (1999) [18], and Módis et al. (2010) [23] confirmed this association, with a greater impact in type 1 DM. McNamara et al. (1998) [17] experimentally demonstrated that changes in aqueous humor glucose directly affect corneal hydration. Other authors found significant correlations between diabetes duration, glycemia, and CCT [20,21]. In type 2 DM, several studies have also reported increased TCC, especially in patients with poorer metabolic control or a longer history of TCC [25,26,27]. In addition, morphological alterations such as pleomorphism and polymegathism have been described, supporting the endothelial pump dysfunction hypothesis [16,18]. However, this systematic review also identified studies in which no significant differences were observed between diabetics and controls. Keoleian et al. (1992) [38] found no variations in TCC between patients with T1DM and controls, while Inoue et al. (2002) [35] and Sudhir et al. (2012) [42] also found no differences in T2DM, even in the presence of retinopathy. Ziadi et al. (2002) [43], after inducing hypoxia with contact lenses, described similar TCC values between diabetics (type 1 and 2) and controls. Other studies agree on the absence of significant differences [40,41]. The heterogeneity of these findings is likely due to methodological limitations, such as the use of different measuring devices, the lack of classification of patients according to diabetes type, HbA1c duration, and publication bias. Even so, most evidence supports the relationship between diabetes and increased TCC, with disease duration and poor glycemic control clearly influencing this relationship. In particular, Olsen et al. (1980), Su et al. (2008), Busted et al. (1981), and Kim and Kim (2021) [14,15,21,39] suggest that an HbA1c > 7% could be associated with an increased risk of endothelial damage and complications during intraocular procedures. Regarding the quantitative results reported in this study, the highest range of CCT was between 10 and 25 years of age, but when these values were compared in the five age ranges evaluated, there were no statistically significant differences associated with this relationship. Regarding HbA1c levels, there are no statistically significant differences in any range with regard to an increase in CCT. These statistical data demonstrate that CCT is theoretically associated with diabetes, but the relationship is not very important to be able to attribute it to any of these factors.

These findings may be explained by the pathophysiological mechanisms inherent to diabetes: chronic hyperglycemia induces sorbitol accumulation, which disrupts endothelial pump function and increases corneal permeability [44]. These changes promote corneal edema and thickening, which explains the increased TCC reported in multiple studies. Therefore, the corneal endothelium in diabetes is subjected to increased metabolic stress, with reduced functional reserve and morphological alterations that contribute to the progression of diabetic keratopathy. In this context, TCC monitoring could play a role in clinical practice as a surrogate marker of corneal damage in diabetic patients. One of the main concerns with the analysis of diabetes duration is the fact that it is very difficult to determine when the diabetes really begins. It is known that cross-sectional studies are more likely to recruit long-standing undiagnosed cases of diabetes as “incident” compared with longitudinal studies [47]. In addition, it has also been determined that, for example, 46% of all adults in California are estimated to have prediabetes or undiagnosed diabetes, and that current diagnostic criteria may not detect high blood sugar levels until the disease has progressed to a later stage [48]. It is also known that diabetes patients with higher CCT values present lower corneal endothelial cell density values. A similar concern may be related to the differences in the CCT values based on the function of the measurement techniques used, the use of anesthetic eye drops, etc. In fact, we have studied all of these concerns in previous articles [49,50,51,52,53,54,55,56,57].

## 5. Limitations

This study has some important limitations that should be considered when interpreting the results. First, a high methodological heterogeneity was identified among the included studies, especially in relation to the devices used to measure CCT, such as ultrasonic pachymeters, Scheimpflug chambers, and specular microscopy, which could affect the comparability of the data. Second, many studies did not adequately classify diabetic patients according to type of diabetes, duration of disease, or HbA1c levels, which makes it difficult to establish clear associations between these variables and CCT values. In addition, some studies did not report relevant information such as sex, age, or comorbidities, which introduces confounding biases.

## 6. Conclusions

The findings of this systematic review and meta-analysis show that patients with DM, both type 1 and type 2, present structural and functional alterations in the cornea, with increased CCT being one of the most frequently reported changes. Although the results were heterogeneous and some studies did not show statistically significant differences, most research suggests that poor glycemic control, especially elevated HbA1c levels, and longer disease duration are associated with morphological alterations, such as increased CCT, polymegathism, pleomorphism, and decreased endothelial cell density. These structural modifications not only reflect the vulnerability of the corneal endothelium to metabolic stress but also represent a potential clinical risk during intraocular procedures, such as cataract and corneal surgery. Therefore, the importance of incorporating periodic evaluation of the CCT and the state of the corneal endothelium as part of the comprehensive ophthalmological follow-up in diabetic patients, especially in those with poor metabolic control or long-term disease, is highlighted. Future research with homogeneous methodology and control of key clinical variables, such as type of diabetes, duration of the disease, and HbA1c levels, could contribute to clarifying more precisely the magnitude and clinical impact of these corneal alterations.

## Figures and Tables

**Table 1:** Studies included in this systematic review. NA: not available; HbA1c: glycosylated hemoglobin; CCT: central corneal thickness; M: male; F: female; OAM: oral antidiabetic medication; OHA: oral hypoglycemic agents; OHT: oral hypoglycemic therapy; OAA: oral antidiabetic agents; PDM: parenteral antidiabetic medication.

Study	Type of DM	Geographical Region	Medication	Sample Size	Study Quality	Gender	Age (years)	Diabetes Duration (Years)	HbA1c Levels (%)	Pachymetry	CCT Values (μm)
Olsen et al. (1980) [15]	1	Finlandia	NA	81	4	NA	NA	15	NA	NA	561 ± 27
Busted et al. (1981) [14]	1	Dinamarca	NA	81	4	NA	30	15	7.5	Haag-Streit pachymeter	544 ± 28
Schultz et al. (1984) [40]	2	EE.UU.	NA	25	3	NA	40 to 49	21	NA	Digital pachymeter	540 ± 20
							50 to 59				530 ± 70
							60 to 69				540 ± 60
							70 to 79				540 ± 80
	1			17			20 to 29	>10			540 ± 80
							30 to 39				540 ± 10
							40 to 59				540 ± 80
Keoleian et al. (1992) [38]	1	EE.UU.	NA	28	3	NA	33 ± 12.0	22 ± 11	10 ± 1.4	Specular microscopy	560 ± 20
Larsson et al. (1996) [16]	1	Suecia	Insulin	49	3	NA	36 ± 12.0	20 ± 11	10.4 ± 2.1	Specular microscopy	580 ± 50
	2		Insulin, OAA, diet	60			60 ± 10.0	13 ± 8.0	9.9 ± 2.1		570 ± 50
McNamara et al. (1998) [17]	1	EE.UU.	Insulin	42	4	23 M, 19 F	39.6 ± 8.8	22.1 ± 10.1	10.2 ± 1.52	Haag-Streit pachymeter	524
Roszkowska et al. (1999) [18]	1	Italia	Insulin	70	2	75 M, 62 F	29.76 ± 3.43	15.3 ± 1.2	<9.5	Specular microscopy	580 ± 20
	2		OHT	23			49.6 ± 6.16	17.2 ± 2.2			570 ± 20
Rosenberg et al. (2000) [19]	1	Finlandia	Insulin	32	4	NA	45.2 ± 10.0	25.9 ± 8.1	NA	In vivo confocal microscopy	576.9 ± 48
Inoue et al. (2002) [35]	2	Japón	NA	196	2	NA	65.5 ± 7.5	9.1 ± 8.2	6.9 ± 1.3	Ultrasound pachymeter	538 ± 36
Ziadi et al. (2002) [43]	1	France	NA	6	3	4 M, 2 F	36 ± 9.0	14 ± 9.0	8.2 ± 1.7	Ultrasound pachymeter	560 ± 38
	2			9		2 M, 7 F	55 ± 8.0	9.0 ± 6.0			547 ± 34
Sonmez et al. (2005) [41]	NA	Turquía	Insulin and OHA	18	3	6 M, 12 F	56.17 ± 5.98	8.67	10.58	Corneal pachymetry and ultrasonic biometry	542.89 ± 37.18
Lee et al. (2006) [20]	1	Corea	NA	300	3	153 M, 147 F	57.5 ± 8.5	10.87 ± 5.9	NA	Ultrasonic pachymetry	588.2 ± 2.7
Wiemer et al. (2007) [45]	1	Países Bajos- Europa	Insulin, OAM	102	3	58 M, 44 F	39.96 ± 10.8	21.06 ± 11.7	8.1 ± 1.6	Scheimpflug camera	586 ± 30
	2			101		54 M, 47 F	56.46 ± 7.0	8.8 6 ± 7.5	7.5 ± 1.4		578 ± 30
Su et al. (2008) [21]	NA	Singapur	NA	748	4	NA	62.59 ± 9.36	NA	8.4 ± 2.0	Ultrasonic pachymetry	547.2
Oriowo et al. (2009) [22]	1	Arabia Saudita	Insulin	86	1	NA	53.96 ± 11.9	13.5	NA	Ultrasonic pachymetry	610
Ozdamar et al. (2010) [24]	1	Turquía	NA	245	3	112 M, 133 F	58.4 ± 8.6	10.0 ± 7.7	NA	Ultrasonic pachymetry	564 ± 30
Módis et al. (2010) [23]	1	Hungría	NA	21	2	12 M, 9 F	40.97 ± 15.46	10.88 ± 8.06	8.55 ± 1.83	Specular microscopy	570 ± 40
	2			30		10 M, 20 F	64.36 ± 10.47	13.61 ± 6.50	8.79 ± 2.01		560 ± 30
Sudhir et al. (2012) [42]	2	India	NA	1312	3	695 M, 617 F	54.8 ± 9.5	NA	NA	Ultrasonic pachymetry	524.75 ± 34.52
Storr-Paulsen et al. (2014) [3]	2	Dinamarca	OAM, PDM	235	4	100 M, 135 F	72.1 ± 11.0	NA	7.3 ± 0.2	Non-contact specular microscopy	546 ± 7
Calvo-Maroto et al. (2015) [25]	2	España	NA	157	3	75 M, 82 F	45.5 ± 2.5	0.38 ± 0.12	7.66 ± 0.78	Ultrasonic pachymeter	546 ± 13
							52.2 ± 1.8	10.2 ± 0.8	7.78 ± 0.66		569 ± 11
Briggs et al. (2015) [26]	2	Arabia Saudita	Insulin and OAM	215	1	118 M, 97 F	56.5 ± 11.9	14.9 ± 8.3	NA	HR Pentacam tomography	539.7 ± 33.6
								<10			
								>10			
Sanchis-Gimeno et al. (2017) [27]	2	España	NA	83	3	40 M, 43 F	33.6 ±3.2	5.9 ± 1.2	6.7 ± 0.3	Non-contact scanning-slit corneal topography	567.4 ± 10.9
Altay et al. (2017) [28]	2	Turquía	NA	264	3	NA	NA	0–4	NA	Scheimpflug camera	532.67 ± 39.35
								4–9			524.54 ± 29.07
								>10			537.20 ± 29.36
								0–4		Non-contact specular microscopy	553.13 ± 38.62
								4–9			544.59 ± 33.87
								>10			558.00 ± 35.76
								0–4		Ultrasonic pachymeter	550.65 ± 38.68
								4–9			537.72 ± 36.36
								>10			553.58 ± 40.08
Fernandes et al. (2019) [34]	1	India	NA	100		52 M, 48 F	12.22 ± 2.82	3.91 ± 1.65	5.8 ± 15.8	NA	525.16 ± 33.14
Chowdhury et al. (2021) [33]	2	India	NA	262		138 M, 124 F	53.26 ± 6.24	7.29 ± 6.00	6.97 ± 0.99	Non-contact specular microscopy	514.54 ± 38.17
Kim and Kim (2021) [39]	2	Corea	NA	1411		597 M, 814 F	65.6 ± 11.1	10.8 ± 8.7	7.54 ± 1.78	Non-contact specular microscope and a Pentacam Scheimpfug camera.	551.80 ± 34.10
Jha et al. (2022) [36]	2	India	NA	1188		NA	62.17 ± 9.49	8.1 ± 5.5	7.5 ± 1.7	Specular microscope with an auto-tracking system.	522.1 ± 36.6
Kan et al. (2023) [37]	2	Malasia	NA	184		184 F	28 ± 4.2	0–9 months	6%	Topcon SP-2000P non-contact specular microscope, and ultrasonography	552.28 ± 22.5
Zulhisham et al. (2023) [44]	1	Malasia	NA	76		37 M, 39 F	14.02 ± 2.66	5.13 ± 2.12	10.68 ± 2.49	Optical coherence tomography	542.18 ± 20.40

**Table 2:** The relationship between the duration of diabetes mellitus (years) and central corneal thickness (CCT).

Author	Diabetes Duration (Years)	CCT
Olsen et al. (1980) [15]	15	561
Busted et al. (1981) [14]	15	544
Schultz et al. (1984) [40]	21	537.5
Keoleian et al. (1992) [38]	22	560
Larsson et al. (1996) [16]	16.5	575
McNamara et al. (1998) [17]	22.1	524
Roszkowska et al. (1999) [18]	16.25	575
Rosenberg et al. (2000) [19]	25.9	576.9
Inoue et al. (2002) [35]	9.1	538
Ziadi et al. (2002) [43]	11.5	553.5
Sonmez et al. (2005) [41]	8.67	542.89
Lee et al. (2006) [20]	10.87	588.2
Wiemer et al. (2007) [45]	14.96	582
Su et al. (2008) [21]	NA	547.2
Oriowo et al. (2009) [22]	13.5	610
Ozdamar et al. (2010) [24]	10.0	564
Módis et al. (2010) [23]	12.245	565
Sudhir et al. (2012) [42]	NA	524.75
Storr-Paulsen et al. (2014) [3]	NA	546
Calvo-Maroto et al. (2015) [25]	5.29	557.5
Briggs et al. (2015) [26]	14.9	539.36
Sanchis-Gimeno et al. (2017) [27]	5.9	567.4
Altay et al. (2017) [28]	0–10	543.49
Fernandes et al. (2019) [34]	3.91	525.16
Chowdhury et al. (2021) [33]	7.29	514.54
Kim and Kim (2021) [39]	10.8	551.80
Jha et al. (2022) [36]	8.1	522.1
Kan et al. (2023) [37]	1.0	552.28
Zulhisham et al. (2023) [44]	5.13	542.18

**Table 3:** Mean and standard deviation of CCT according to the ranges of duration of diabetes mellitus.

Range Diabetes Duration (Years)	Mean/SD of CCT	Difference Mean 95%
1–5.9	548.90/14.39	*p* = 0.412
6.0–10.9	545.63/21.73	
11–15.9	564.98/22.55	
16–20.9	575/0	
21–25.9 (4)	549.6/20.34	

**Table 4:** CCT values reported according to glycosylated hemoglobin (HbA1c) levels.

Author	HbA1c Levels (%)	CCT
Olsen et al. (1980) [15]	NA	561
Busted et al. (1981) [14]	7.5	544
Schultz et al. (1984) [40]	NA	537.5
Keoleian et al. (1992) [38]	10	560
Larsson et al. (1996) [16]	10.15	575
McNamara et al. (1998) [17]	10.2	524
Roszkowska et al. (1999) [18]	<9.5	575
Rosenberg et al. (2000) [19]	NA	576.9
Inoue et al. (2002) [35]	6.9	538
Ziadi et al. (2002) [43]	8.2	553.5
Sonmez et al. (2005) [41]	10.58	542.89
Lee et al. (2006) [20]	NA	588.2
Wiemer et al. (2007) [45]	7.86	582
Su et al. (2008) [21]	8.4	547.2
Oriowo et al. (2009) [22]	NA	610
Ozdamar et al. (2010) [24]	NA	564
Módis et al. (2010) [23]	8.67	565
Sudhir et al. (2012) [42]	NA	524.75
Storr-Paulsen et al. (2014) [3]	7.3	546
Calvo-Maroto et al. (2015) [25]	7.72	557.5
Briggs et al. (2015) [26]	NA	539.37
Sanchis-Gimeno et al. (2017) [27]	6.7	567.4
Altay et al. (2017) [28]	NA	543.49
Fernandes et al. (2019) [34]	5.8	525.16
Chowdhury et al. (2021) [33]	6.97	514.54
Kim and Kim (2021) [39]	7.54	551.80
Jha et al. (2022) [36]	7.5	522.1
Kan et al. (2023) [37]	6	552.28
Zulhisham et al. (2023) [44]	10.68	542.18

**Table 5:** Characteristics, months of diabetes mellitus, HbA1c, and CCT level.

	Subjects	Average Age	Months of Diabetes Mellitus (2)	HbA1c	Level of CCT
N	29	29	29	29	29
Mean	271	42.7	131	5.66	553
Std. error mean	71.5	3.83	15.4	0.754	4.07
95% CI mean lower bound	124	34.8	99.6	4.11	544
95% CI mean upper bound	417	50.5	163	7.20	561
Median	109	48.2	130	7.30	552
Standard deviation	385	20.6	82.8	4.06	21.9
Variance	148,377	425	6863	16.5	480
Range	1396	72.1	311	10.7	95.5
Minimum	15	0.00	0.00	0.00	515
Maximum	1411	72.1	311	10.7	610
Skewness	2.24	−0.911	0.222	−0.545	0.491
Std. error skewness	0.434	0.434	0.434	0.434	0.434
Kurtosis	4.00	−0.072	−0.388	−1.37	0.303
Std. error kurtosis	0.845	0.845	0.845	0.845	0.845
Shapiro-Wilk W	0.620	0.897	0.969	0.806	0.975
Shapiro-Wilk p	<0.001	0.008	0.532	<0.001	0.708

Note: The CI of the mean assumes sample means follow a t-distribution with N − 1 degrees of freedom.

**Table 6:** Risk of bias, including studies assessed with Robins-I.

Author	Confounding	Selection	Measurement of Intervention	Missing Data	Measurement of Outcomes	Reported Result	Overall
Olsen et al., 1980 [15]	Low	High (only pregnant women and a small sample)	Low	Not mentioned	High	Not determined	High
Busted et al., 1981 [14]	Low	Low	Low	Low	Low	Low	High
Schultz et al., 1984 [40]	Low	High (only patients with DM, selection based on age, same duration of diabetes, and those who are insulin dependent)	Low	Low	Low	Low	High
Keoleian et al., 1992 [38]	Low	High (small sample)	Low	Not mentioned	Low	Low	High
Larsson et al., 1996 [16]	High	High (patients with DM I and II, and only 30 patients without diabetes)	Low	Low	Low	Low	Low
McNamara et al., 1998 [17]	Low	Low	Low	High	Low	Low	Low
Roszkowska et al., 1999 [18]	Low	Low	Low	Low	High	Low	Low
Rosenberg et al., 2000 [19]	Low	High (low number in the control group)	Low	Low	Low	Low	Low
Inoue et al., 2002 [35]	High	Low	Low	Low	Low	Low	Low
Ziadi et al., 2002 [43]	High	High (with controlled information)	Low	Low	Low	Low	High
Sonmez et al., 2005 [41]	Low	High (only patients with DM)	Low	Low	Low	Low	High
Lee et al., 2006 [20]	Low	Low	Low	Low	Low	Low	High
Wiemer et al., 2007 [45]	Low	Low	Low	Low	High	Low	Low
Su et al., 2008 [21]	High	High (only people with DM)	High	Low	High	Low	Low
Oriowo et al., 2009 [22]	Low	Low	Low	Low	Low	Low	Low
Ozdamar et al., 2010 [24]	Low	Low	Low	Low	Low	Low	Low
Módis et al., 2010 [23]	Low	High (only people with DM)	Low	Low	Low	Low	Low
Sudhir et al., 2012 [42]	High	High (Only people with DM)	Low	Low	Low	Low	High
Storr-Paulsen et al., 2014 [3]	Low	Low	Low	Low	Low	Low	Low
Calvo-Maroto et al., 2015 [25]	Low	Low	Low	Low	High	Low	Low
Briggs et al., 2015 [26]	Low				Low		Low
Sanchis-Gimeno et al., 2017 [27]	Low	Low	Low	Low	Low	Low	Low
Altay et al., 2017 [28]	High	Low	Low	Low	Low	Low	Low
Fernandes et al., 2019 [34]	Low	Low	Low	The participants are not mentioned, only the data.	Low	Low	Low
Chowdhury et al., 2021 [33]	Low	Low	Low	Low	Low	Low	High
Kim and Kim 2021 [39]	Low	Low	Low	Low	Low	The procedure is not explained very well with respect to the conclusion.	High
Jha et al., 2022 [36]	High	Low	Low	Not mentioned the sample size, only the results	Low	Low	Low
Kan et al., 2023 [37]	Low	Low	Low	Low	Low	Low	Low
Zulhisham et al., 2023 [44]	Low	High (small sample)	Low	Low	Low	Low	Low

## Supplementary Materials

The supporting information can be downloaded at: https://www.mdpi.com/article/10.3390/ijms26178695/s1.
