# Supplementary material for: The Effect of Diabetes Mellitus on Central Corneal Thickness Values: A Systematic Review and Meta-Analysis
Source: Int J Mol Sci. 2025 Sep 6;26(17):8695. doi: 10.3390/ijms26178695 (PMC12429604; doi:10.3390/ijms26178695)
Supplement: Supplementary file 1 [file ijms-26-08695-s001.zip › ijms-3792924-supplementary.pdf]

Table S1. Details of the search strategy.

Database	Search strategy	Results	
		20-03-25	15-08-2025
Medline	Central corneal thickness OR CCT AND Diabetes mellitus OR DM2 OR DM1 AND Corneal disease ((((("central"[All Fields] OR "centrally"[All Fields] OR "centrals"[All Fields]) AND ("cornea"[MeSH Terms] OR "cornea"[All Fields] OR "corneal"[All Fields]) AND ("thick"[All Fields] OR "thickness"[All Fields] OR "thicknesses"[All Fields])) OR "CCT"[All Fields]) AND ("diabetes mellitus"[MeSH Terms] OR ("diabetes"[All Fields] AND "mellitus"[All Fields]) OR "diabetes mellitus"[All Fields])) OR "DM2"[All Fields] OR "DM1"[All Fields]) AND ("corneal diseases"[MeSH Terms] OR ("corneal"[All Fields] AND "diseases"[All Fields]) OR "corneal diseases"[All Fields] OR ("corneal"[All Fields] AND "disease"[All Fields]) OR "corneal disease"[All Fields])	301	301
Wos	Central corneal thickness OR CCT AND Diabetes mellitus OR DM2 OR DM1 AND Corneal disease	100	101
CINAHL	Central corneal thickness OR CCT AND Diabetes mellitus OR DM2 OR DM1 AND Corneal disease	10	10
SCOPUS	Central corneal thickness OR CCT AND Diabetes mellitus OR DM2 OR DM1 AND Corneal disease	40	40
Google Scholar	Central corneal thickness OR CCT AND Diabetes mellitus OR DM2 OR DM1 AND Corneal disease	84	84
	Total	535	536

* All searches were carried out on August 15, 2025.
